# Supplementary material for: Structural basis of membrane potential coupled vectorial CO₂ hydration by the DAB2 complex in chemolithoautotrophs
Source: Nat Commun. 2026 May 5;17:4071. doi: 10.1038/s41467-026-72558-7 (PMC13144422; doi:10.1038/s41467-026-72558-7)
Supplement: Supplementary file 1 — Supplementary Information [file 41467_2026_72558_MOESM1_ESM.pdf]

Supplementary information

Structural Basis of Membrane Potential Coupled Vectorial CO_2 Hydration by the DAB2 Complex in Chemolithoautotrophs

Yat Kei Lo, Michael Seletskiy, Stefan Bohn, Darja Deobald, Timo Glatter, Sven T. Stripp, Jan M. Schuller

List of supplementary information

Supplementary Figures

Supplementary Figure 1. Construction and purification of *Dab2*.
Supplementary Figure 2. Cryo-EM processing workflow for *Dab2-*CO_2.
Supplementary Figure 3. Cryo-EM processing workflow for *Dab2-*HCO_3^-.
Supplementary Figure 4. Cryo-EM processing workflow for Dab2-Ambient.
Supplementary Figure 5. Representative density fitting.
Supplementary Figure 6. Comparison of *Dab2* under different conditions.
Supplementary Figure 7. DabA2 catalytic domain structurally mimicked β-CAs.
Supplementary Figure 8. Amino acid conservation in DAB2.
Supplementary Figure 9. Interactions between DabA2 and DabB2.
Supplementary Figure 10. Inductively coupled plasma mass spectrometry.
Supplementary Figure 11. Protein expression level of Dab2 variants determined by LC-MS.
Supplementary Figure 12. Active site water network.
Supplementary Figure 13. FTIR complete dataset.
Supplementary Figure 14. Absolute spectra of CO_2 in solution with H_2O or dimethyl sulfoxide.
Supplementary Figure 15. Effect of L658 substitution on DAB2 activity.
Supplementary Figure 16. Ligands density fitting along the predicted tunnels.
Supplementary Figure 17. DabB2 Multiple sequence alignment.
Supplementary Figure 18. DAB2 activity is independent of sodium.
Supplementary Figure 19. Putative proton pathway water molecules.

Supplementary Tables

Supplementary Table 1. Cryo-EM data collection, refinement and validation statistics.
Supplementary Table 2. Polar interactions between DabA2 and DabB2.

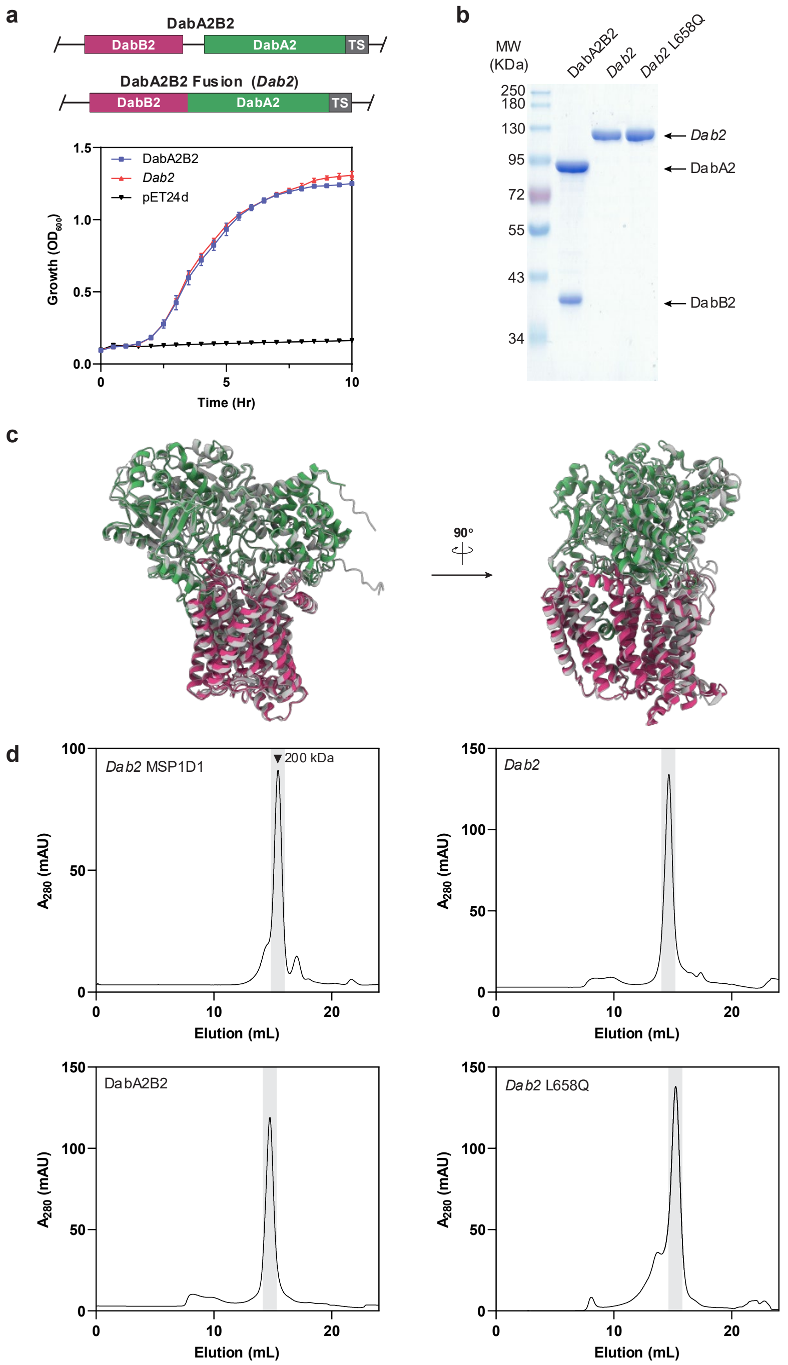

Supplementary Figure 1. Construction and purification of *Dab2*. a) Construct designs for purification of the wild-type DabA2B2 complex and the fusion variant (*Dab2*). TS: twin-strep tag. Both DabA2B2 and *Dab2* were able to complement CA deficient *Escherichia coli* under low CO_2 condition (0.04%) to a similar extend. Strain transformed with empty plasmid pET24d was used as a negative control. Data points and error bars represent means and standard deviations, respectively (n = 4 biological replicates). **b)** SDS-PAGE of purified proteins after size exclusion chromatography. **c)** Superposition of AlphaFold predicted model of DabA2B2 (gray) on the *Dab2* fusion protein (colored as in Fig. 1; RMSD = 1.17 Å). The predicted model is available as Supplementary Data. **d)** Size-exclusion chromatograms of the purified proteins in 0.03% DDM and after nanodisc (MSP1D1) reconstitution. Fractions under the gray area were collected.

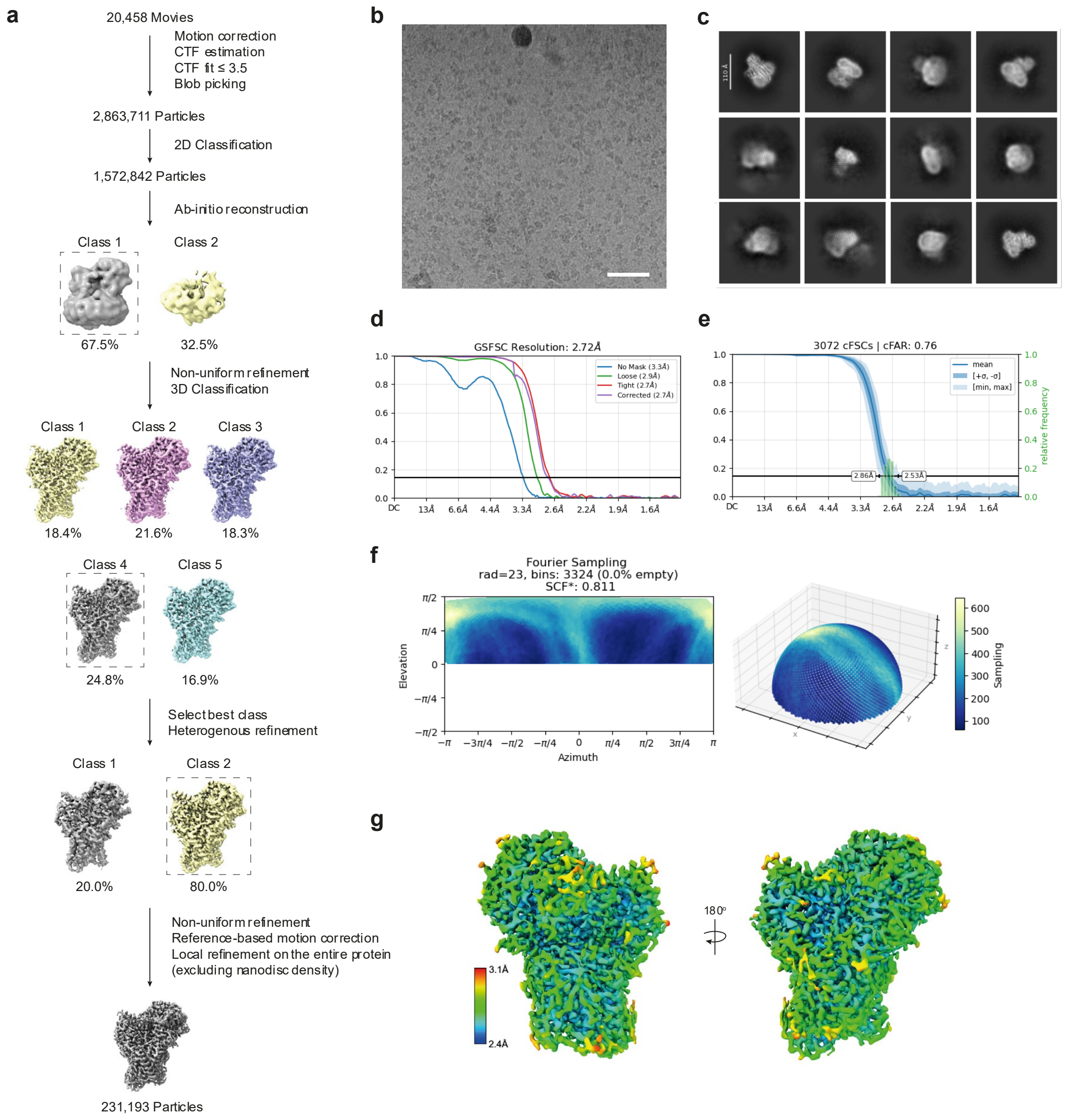

Supplementary Figure 2. Cryo-EM processing workflow for *Dab2-CO₂*. **a)** Overview of the data processing pipeline (see Methods). **b)** Representative micrograph acquired on Falcon 4i Detector (scale bar, 50 nm). 20,458 micrographs were collected. **c)** Representative 2D class averages. **d)** Fourier shell correlation (FSC) curves before and after applying masks. Global resolution is reported at FSC = 0.143. **e)** Directional resolution and **f)** Sampling Compensation Factor (SCF*) of the final reconstruction. **g)** Local resolution (Å) presented in front and back views.

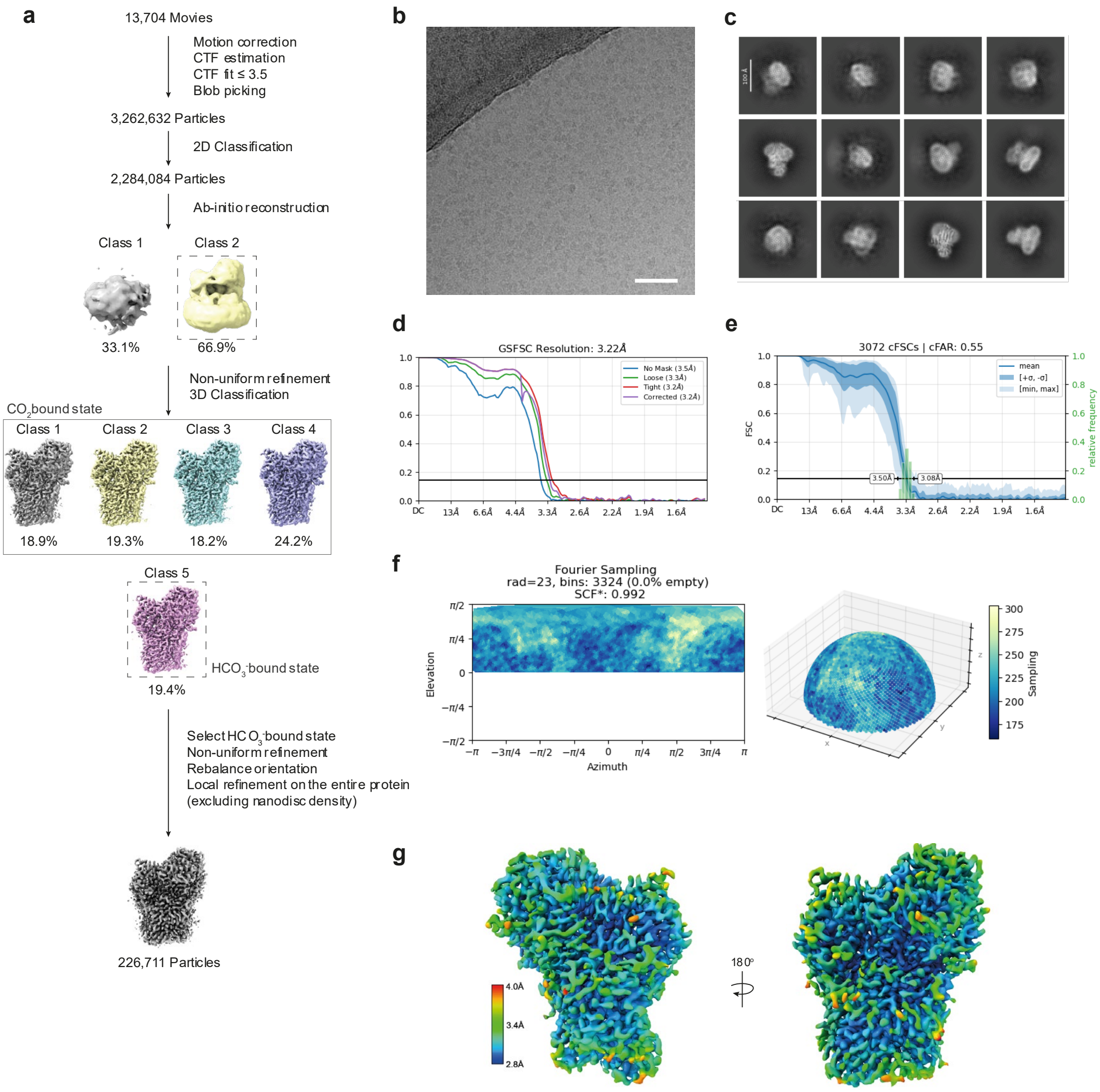

Supplementary Figure 3. Cryo-EM processing workflow for *Dab2-HCO₃⁻*. a) Overview of the data processing pipeline (see Methods). **b)** Representative micrograph acquired on Falcon 4i Detector (scale bar, 50 nm). 13,704 micrographs were collected. **c)** Representative 2D class averages. **d)** Fourier shell correlation (FSC) curves before and after applying masks. Global resolution is reported at FSC = 0.143. **e)** Directional resolution and **f)** Sampling Compensation Factor (SCF*) of the final reconstruction. **g)** Local resolution (Å) presented in front and back views.

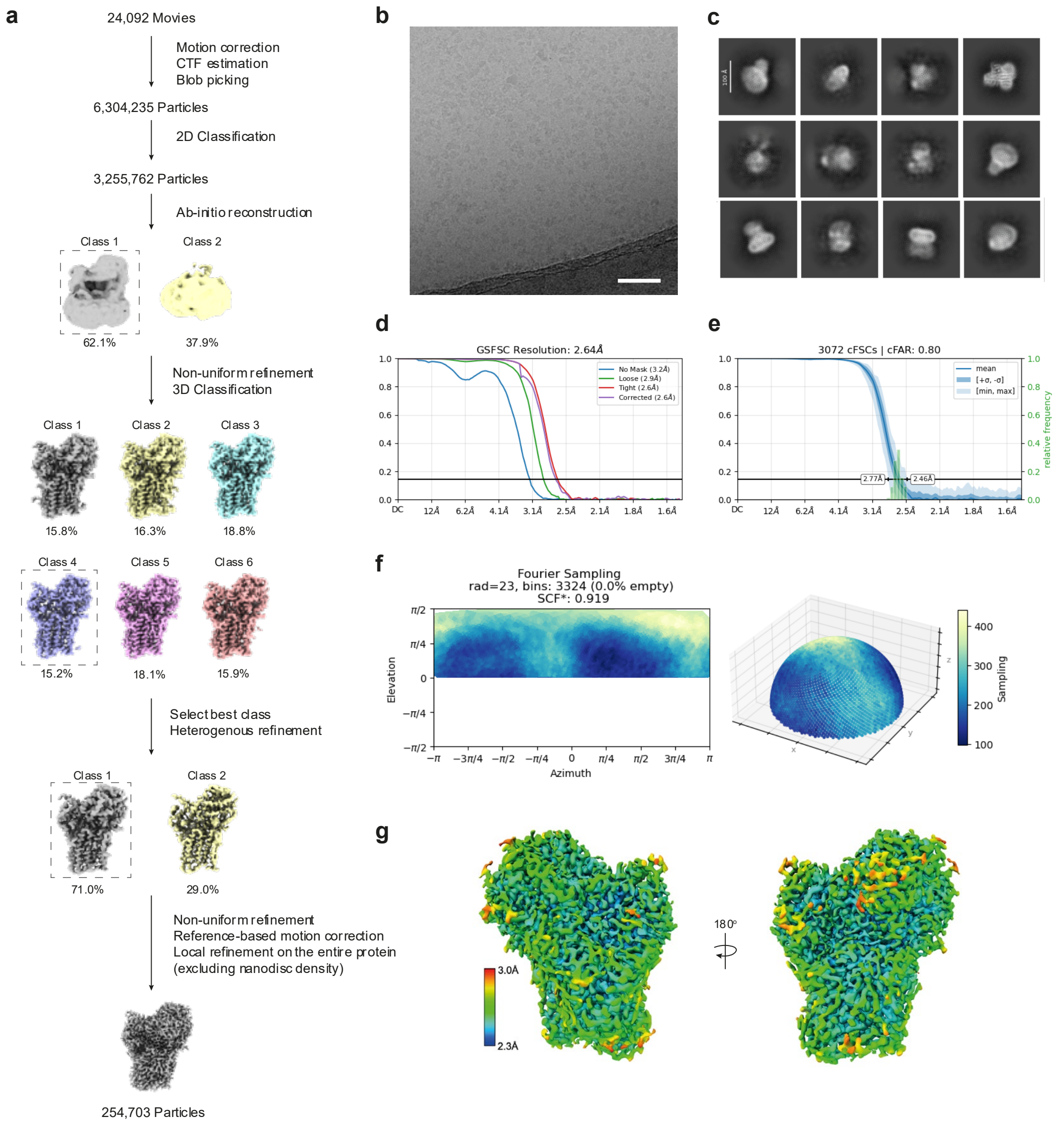

Supplementary Figure 4. Cryo-EM processing workflow for Dab2-Ambient. a) Overview of the data processing pipeline (see Methods). **b)** Representative micrograph acquired on Falcon 4i Detector (scale bar, 50 nm). 24,092 micrographs were collected. **c)** Representative 2D class averages. **d)** Fourier shell correlation (FSC) curves before and after applying masks. Global resolution is reported at FSC = 0.143. **e)** Directional resolution and **f)** Sampling Compensation Factor (SCF*) of the final reconstruction. **g)** Local resolution (Å) presented in front and back views.

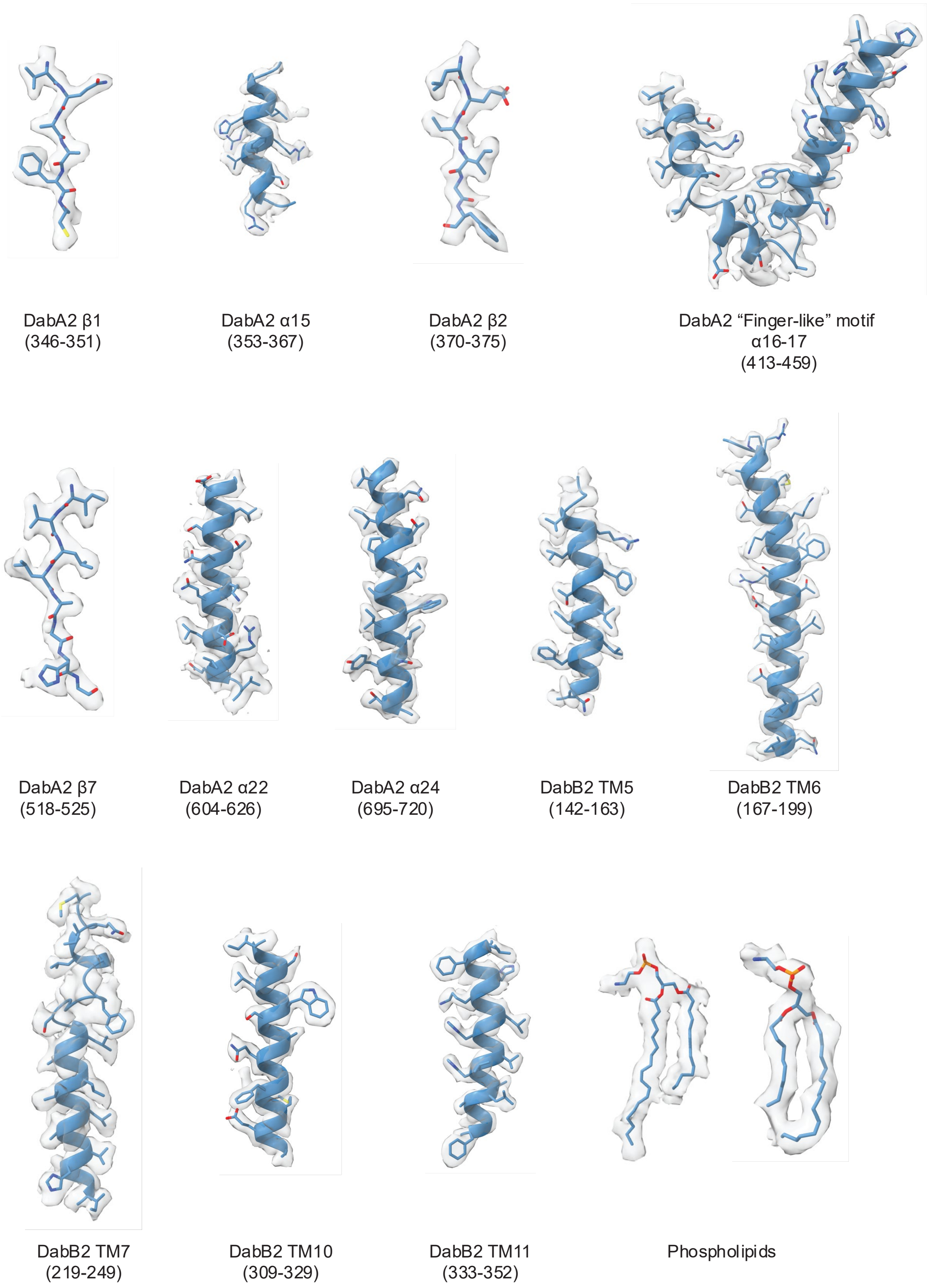

Supplementary Figure 5. Representative density fitting. Density fitting of representative structures in DabA2, DabB2 and phospholipids.

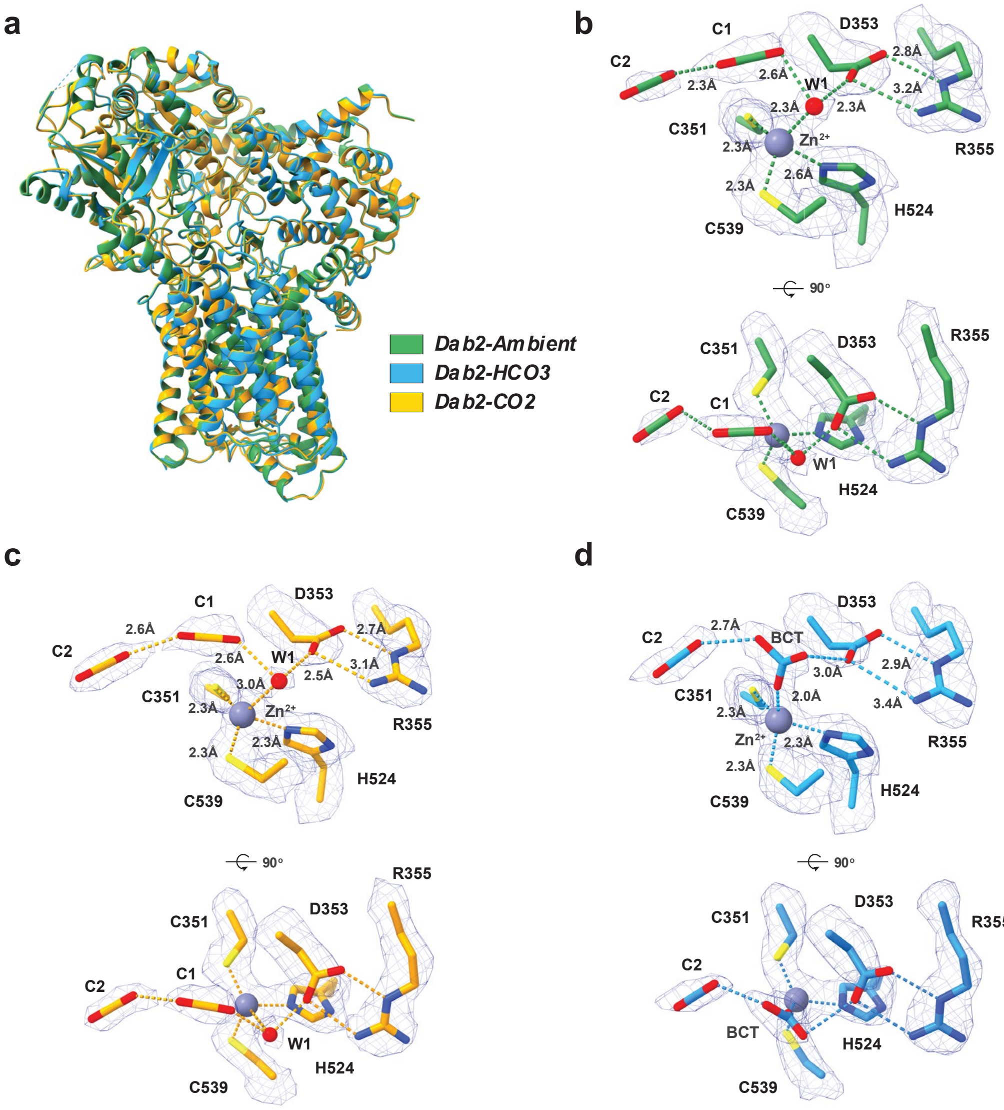

Supplementary Figure 6. Comparison of *Dab2* under different conditions. a) Superposition of *Dab2-ambient* (green), *Dab2-*HCO_3^- (blue), and *Dab2-*CO_2 (yellow). **b-d)** Active site architecture and the density fitting of *Dab2* under different conditions, colored as in **a**. Maps are displayed at a comparable threshold (*Dab2-Ambient*: 7 σ; *Dab2-*CO_2: 9.2 σ; *Dab2-*HCO_3^-: 6.5 σ). Dashes illustrate interactions between atoms and ions.

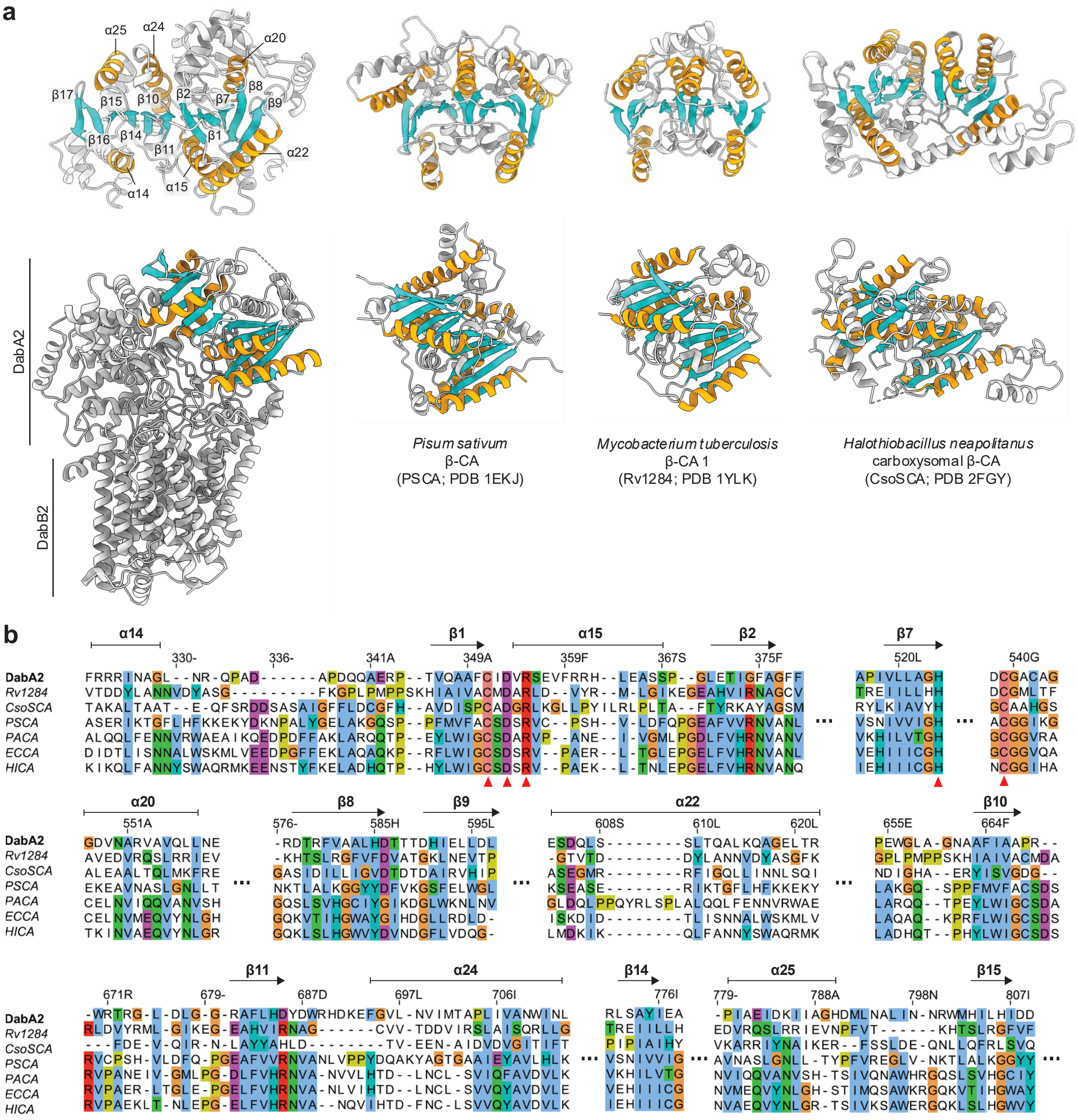

Supplementary Figure 7. DabA2 catalytic domain structurally mimicked β-CAs. a) Structural comparison between *Dab2* and representative β-CAs, shown in top and side views. DabA2 catalytic domain was characterized by two β-CA-like Rossmann folds, colored by secondary structure (orange: α-helix; cyan: β-sheet). The first fold consisted of four parallel β-sheets in the order of 2-1-3-4 (β2-β1-β7-β8) and an antiparallel sheet β9. β3-β6 formed the scaffold for the "finger-like" motifs (See Fig. 1d). The second fold had an additional anti-parallel β-sheets, arranged in the ordered of β11-β10-β14-β15-β16-β17. β12 and β13 formed part of the interacting interface with DabB2. **b)** Structure-based sequence alignment of DabA2 and β-CAs. Rv1284: *Mycobacterium tuberculosis* β-CA (PDB 1YLK); CsoSCA: *Halothiobacillus neapolitanus* β-CA (PDB 2FGY); PSCA: *Pisum sativum* β-CA (PDB 1EKJ); PACA: *Pseudomonas aeruginosa* β-CA (PDB 5BQ1); ECCA: *E. coli* β-CA (PDB 2ESF) HICA: *Haemophilus influenzae* β-CA (PDB 2A8D). Alignment is visualized in Jalview, and colored by the *Clustal* coloring scheme. Strictly conserved residues are marked by red triangles. DabA2 residues number and secondary structure are shown above the alignment. Un-aligned regions are trimmed and represented by dotted lines.

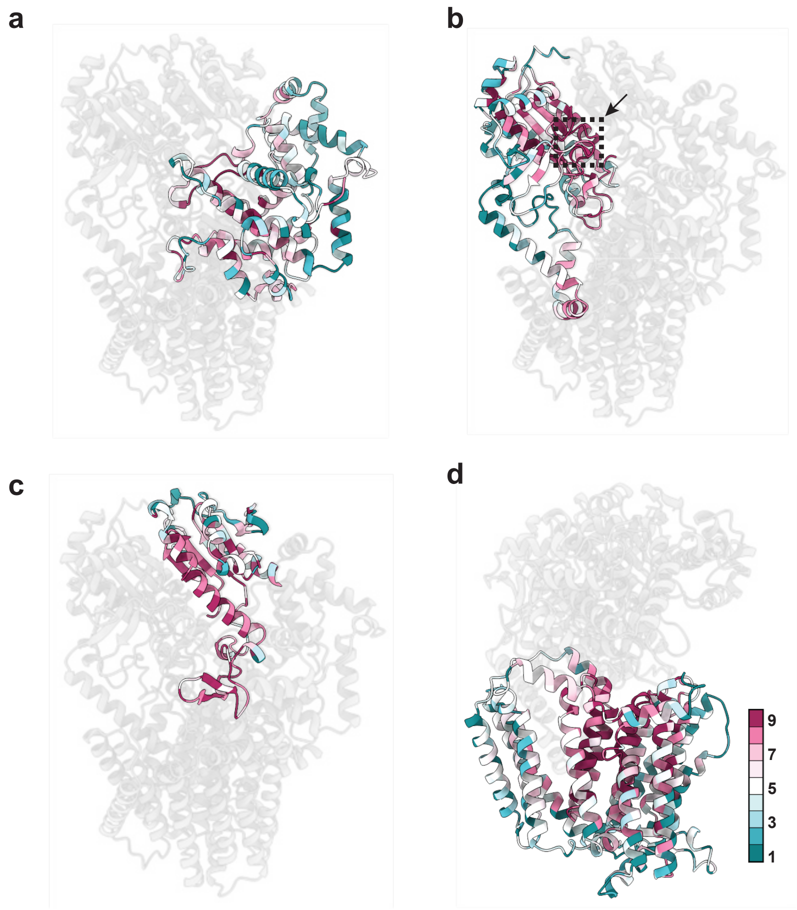

Supplementary Figure 8. Amino acid conservation in DAB2. Sequence conservation of **a)** N-terminal domain, **b)** β-CA-like I domain, **c)** β-CA-like II domain of DabA2 and **d)** DabB2 calculated using the ConSurf server. Residues are colored by conservation level from highest (9) to lowest (1). **b)** Box and arrow indicate position of the active site.

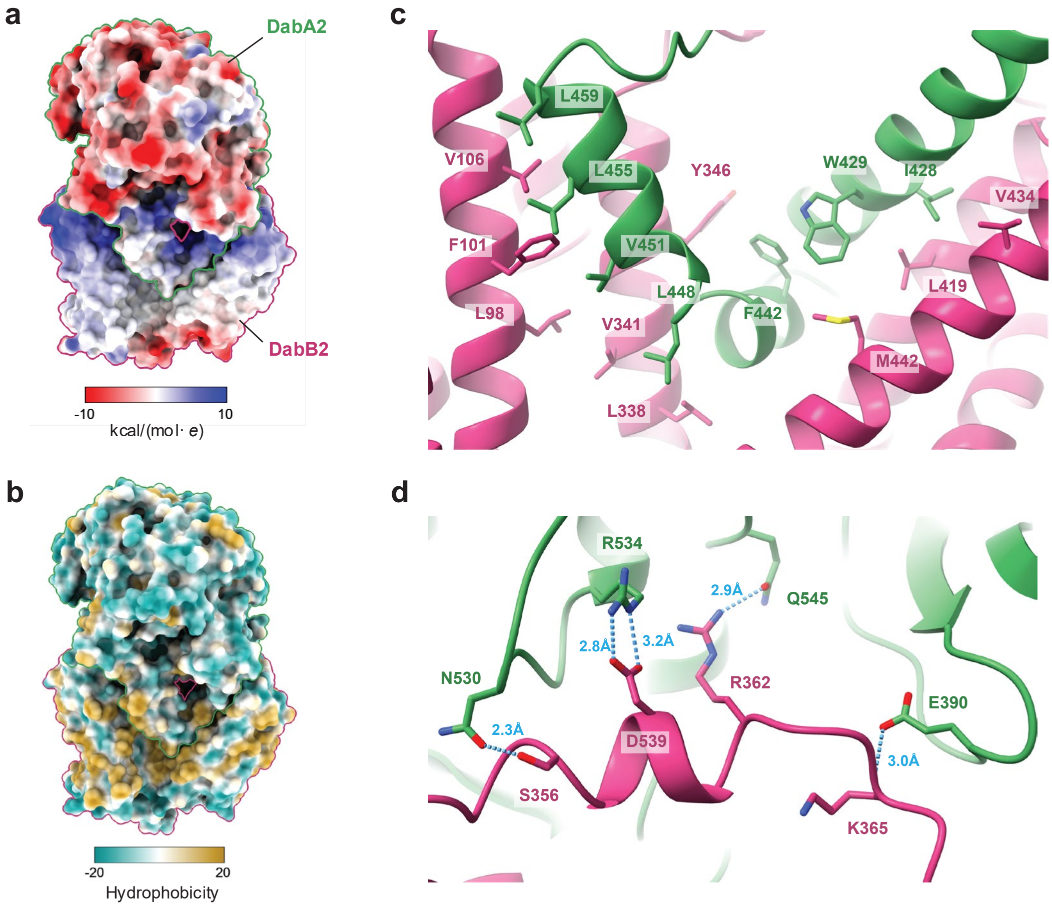

Supplementary Figure 9. Interactions between DabA2 and DabB2. a) Surface coulombic electrostatic potential and **b)** hydrophobicity calculated by ChimeraX. Binding of DabA2 with DabB2 involved interactions at the cytoplasmic interface and hydrophobic interactions between DabB2 transmembrane helixes and DabA2 "finger-like" motif. **c)** Overview of side chain hydrophobic interactions between DabB2 and DabA2 "finger-like" motif. **d)** Representative hydrogen-bonds and salt bridge between DabA2 and DabB2 cytoplasmic helix (α2). See Supplementary Table 2 for the list of polar interactions.

a

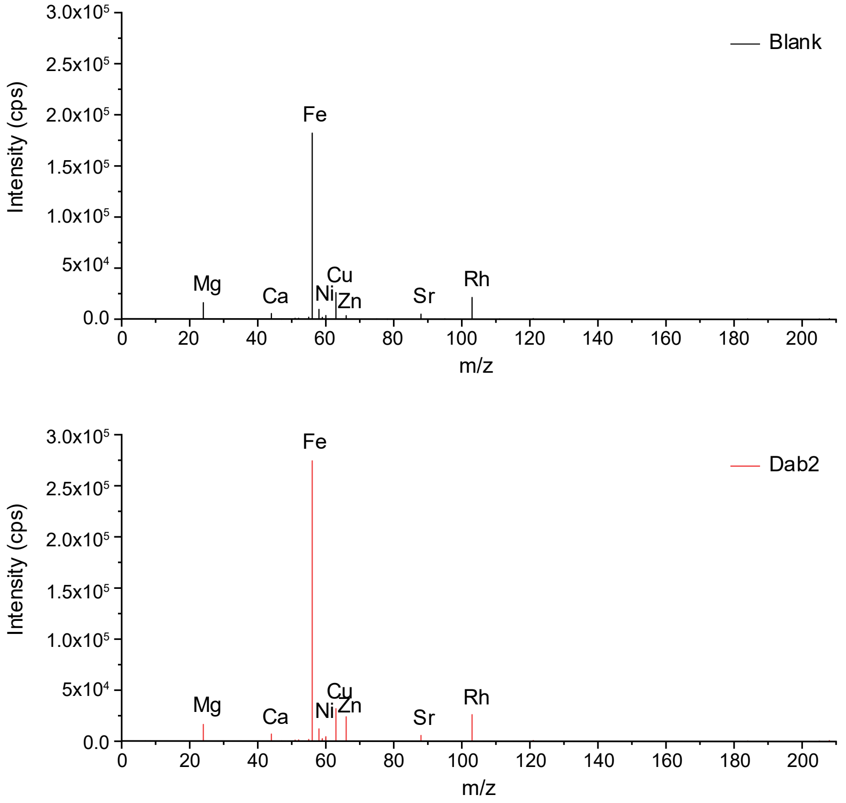

b

Metal ion	Concentration (μM)	SD (μM)	Metal ion / protein	SD
^{66}Zn	29.11	2.73	1.0	0.1
^{24}Mg	0.00	1.33	0.0	0.0
^{44}Ca	4.30	22.62	0.1	0.7
^{55}Mn	0.00	0.14	0.0	0.0
^{56}Fe	0.00	5.29	0.0	0.2
^{58}Ni	0.00	0.20	0.0	0.0
^{59}Co	0.00	0.05	0.0	0.0
^{63}Cu	0.00	0.69	0.0	0.0

Supplementary Figure 10. Inductively coupled plasma mass spectrometry. a) Representative ICP-MS spectra of Dab2 and blank. **b)** Concentration of metal ion detected from 30.6 μM protein and the corresponding metal-to-protein ratio. Means and standard deviations of three technical replicates are reported.

a

	DabA2		DabB2	
Variants	Log2 ratio	q-value	Log2 ratio	q-value
pBAB30	-8.377	0.0002	-5.418	0.0008
DabA2 C351A	0.092	0.8427	-2.761	0.3126
DabA2 D353A	0.149	0.8553	-3.788	0.2346
DabA2D353N	0.087	0.9436	-3.622	0.2940
DabA2 R355A	0.000	0.9823	-3.396	0.1396
DabA2 E444A	-0.107	0.4423	-2.794	0.0310
DabA2 E444Q	-0.056	0.7266	-1.331	0.2949
DabA2 H524A	0.046	0.9800	-0.462	0.6647
DabA2 C539A	0.202	0.8107	-0.402	0.5912
DabB2 I151E	-0.208	0.0896	-1.779	0.1332
DabB2 R182A	-0.695	0.0334	-2.838	0.0330
DabB2 D185A	-0.398	0.0257	-1.116	0.3795
DabB2 K234A	-0.979	0.0020	-0.963	0.5030
DabB2 S256A	-0.553	0.0041	-2.534	0.0307
DabB2 H260A	-0.425	0.0723	-1.944	0.1258
DabB2 K310A	-0.428	0.0228	-3.182	0.0169
DabB2 S316A	-0.117	0.5761	-1.140	0.4088
DabB2 H339A	0.030	0.9664	-2.094	0.1932
DabB2 H343A	-0.029	0.8431	-1.735	0.1908
DabB2 K347A	-0.501	0.0177	-1.223	0.2377

b

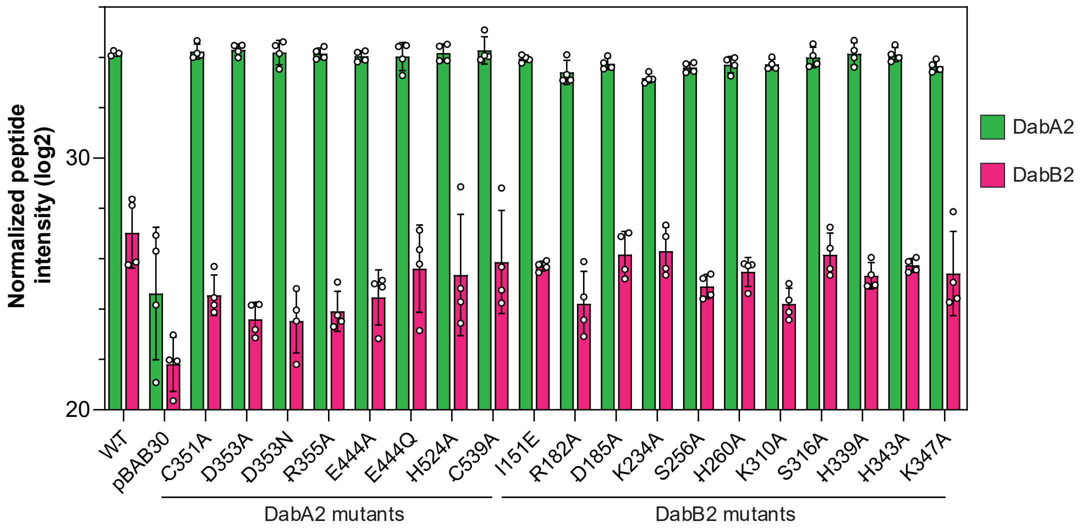

Supplementary Figure 11. Protein expression level of Dab2 variants determined by LC-MS. a) Log2 ratio of the normalized and imputed mean of *Dab2* variants peptide intensity to the wild-type, expressed from the pBAD30 plasmid. See Materials and Methods for details. Lysate from strain carrying the empty pBAD30 plasmid was utilized as a 'non-expressing' negative control. Log2 ratio of zero indicates identical expression level as the wild-type complex. **b)** Sum of peptide intensity for each variant imputed and normalized to the median protein sum of the total intensity distribution. The values were used to obtain panel **a**. Bar heights and error bars represent means and standard deviations, respectively (n = 4 biological replicates). Source data are available via ProteomeXchange with identifier PXD076189.

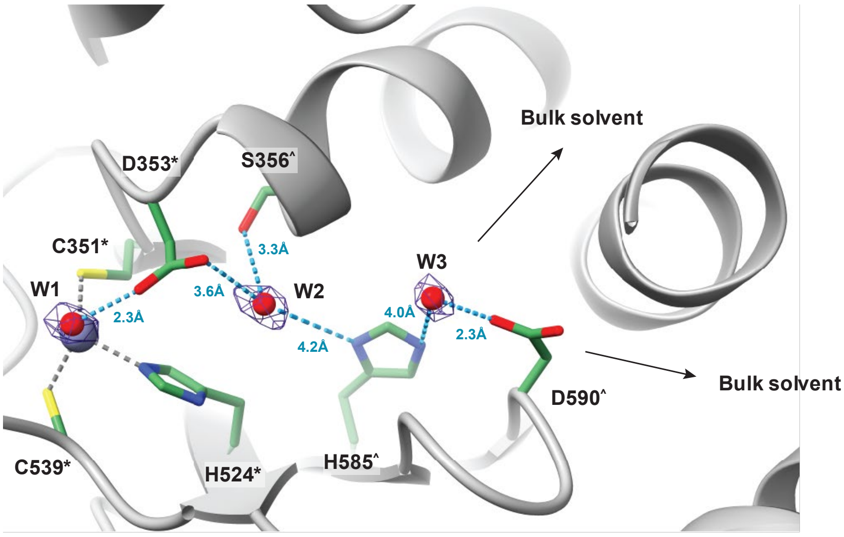

Supplementary Figure 12. Active site water network. CO_2 hydration is initiated by deprotonation of a zinc-bound water into a hydroxide ion. The water network (blue dashes) and charged residues connecting the active site and the bulk solvent may serve as a putative pathway for removing the proton. Densities of water molecules are displayed at 7 σ. Key residues are shown in green. Residues strictly conserved or conserved at ≥ 90% sequence identity among DabA2 homologues are marked by asterisk (*) and circumflex (^) respectively.

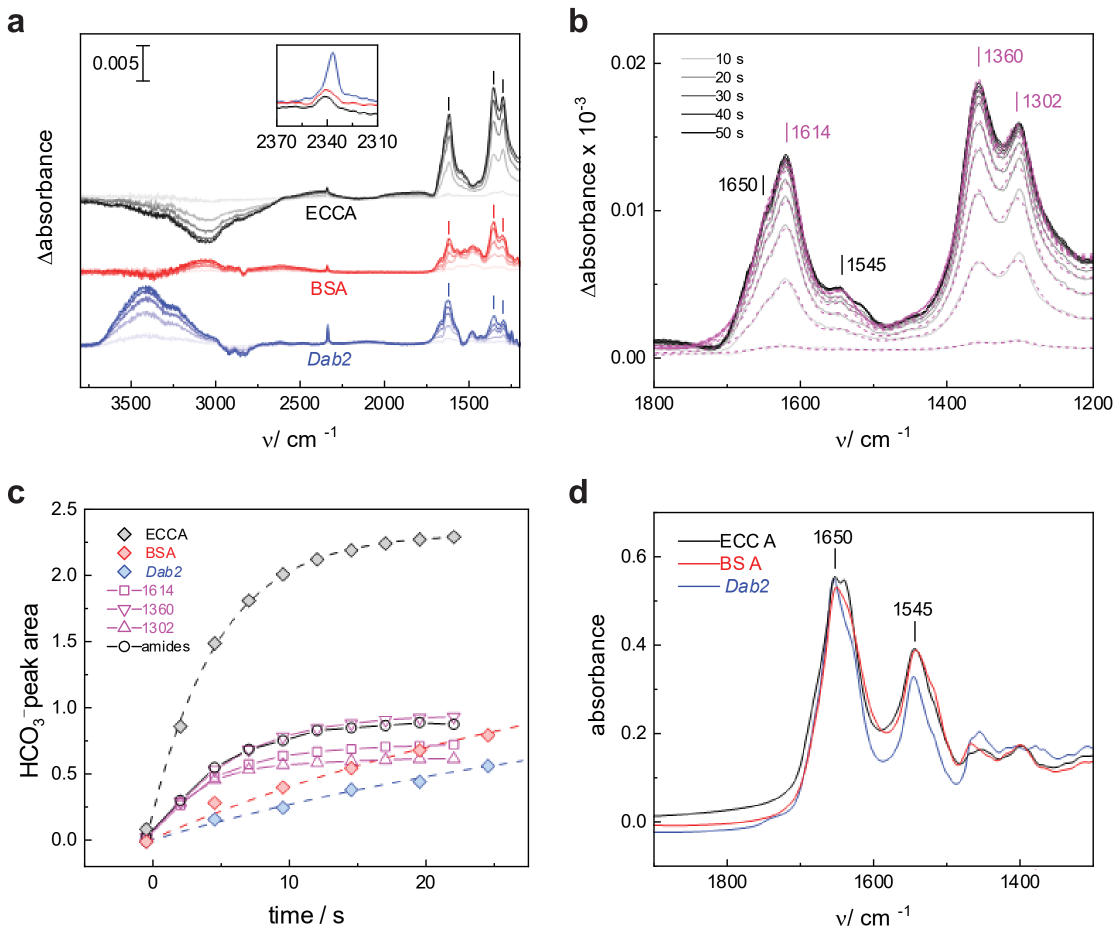

Supplementary Figure 13. FTIR complete dataset. a) Complete "CO_2-minus-N_2" difference spectra for ECCA, BSA, and *Dab2* between 3800 and 1200 cm^{-1}. ECCA shows a characteristic consumption of H_2O at approx. 3000 cm^{-1}, as observed earlier[29]. The positive band at approx. 3400 cm^{-1} in the *Dab2* spectra hint at an unspecific increase of protein film hydration. The inset highlights the CO_2 band as discussed in the main text (Fig. 3). **b)** Exemplary fit of the ECCA difference spectra between 1800–1200 cm^{-1}. The fit (dashed magenta traces) includes positive contributions from HCO_3^- at 1614, 1360, and 1302 cm^{-1} as well as the amide bands (compare panel **d**). **c)** Time traces for the increase of HCO_3^- in ECCA, BSA, and *Dab2* based on these fits. The peak area of the three components at 1614, 1360, and 1302 cm^{-1} (shown exemplary for ECCA, grey traces) are combined to one value ("HCO_3^- peak area") and plotted against time. While ECCA shows a fast logarithmic increase that reaches equilibrium after 20 s, BSA and Dab2 display a slow increase that fits the early, linear phase of product increase. **d)** Comparison of the amide I (1650 cm^{-1}) and amide II (1545 cm^{-1}) bands of the ECCA, BSA, and *Dab2* protein films. The comparable amide ratio suggests similar hydration levels and protein concentrations in each film. Source data are provided as a Source Data file.

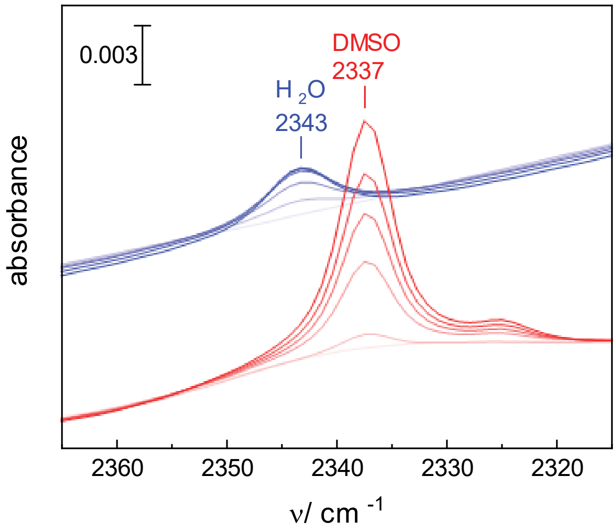

Supplementary Figure 14. Absolute spectra of CO_2 in solution with H_2O or dimethyl sulfoxide (DMSO). Time traces for the increase of CO_2 dissolved in H_2O (blue traces) or DMSO (red traces) in the presence of 10% gaseous CO_2. The 6 cm^{-1} difference in the CO_2 marker band reflects the shift from a hydrogen-bonding, protic environment (H_2O, 2342 cm^{-1}) to a hydrophobic, aprotic environment (DMSO, 2337 cm^{-1}). Source data are provided as a Source Data file.

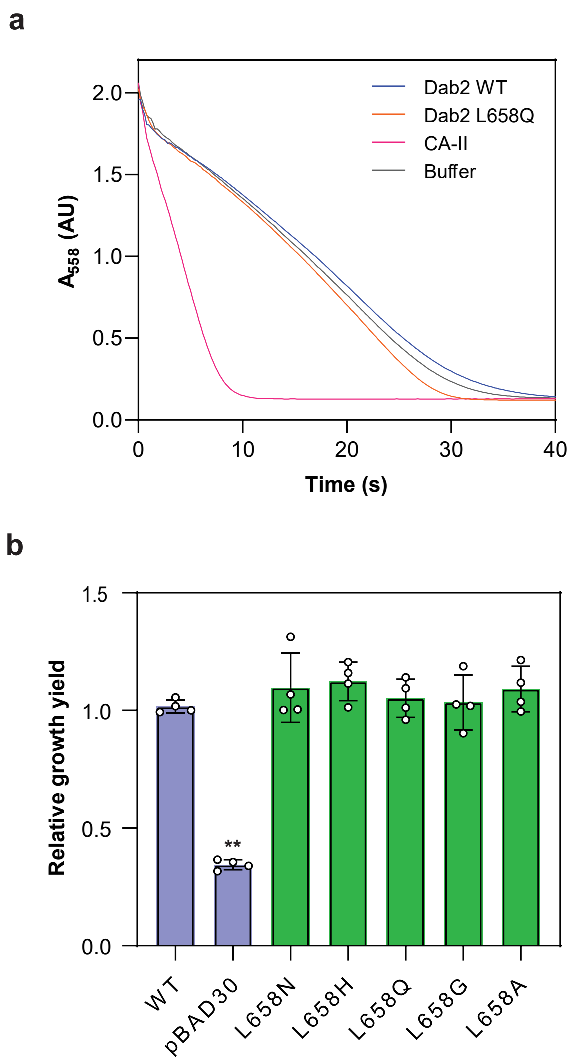

Supplementary Figure 15. Effect of L658 substitution on DAB2 activity. **a)** Representative results of CO_2 hydration activity measured as a function of pH reduction, indicated by phenol red absorbance at 558 nm (n = 3 technical replicates). 5 nM Bovine carbonic anhydrase II (CA-II) was used as a positive control. Both Dab2 WT (500 nM) and L658Q variant (500 nM) showed similar background CO_2 hydration as the negative control (Buffer). **b)** Substitutions of Leu658 did not impair DAB2 ability to complement CA deficient *E. coli*. Bar heights and error bars represent means and standard deviations, respectively (n = 4 biological replicates). "**" Indicates statistically significant difference compared to WT ($P < 0.05$) according to Holm-Bonferroni corrected two-tailed t-test. Source data are provided as a Source Data file.

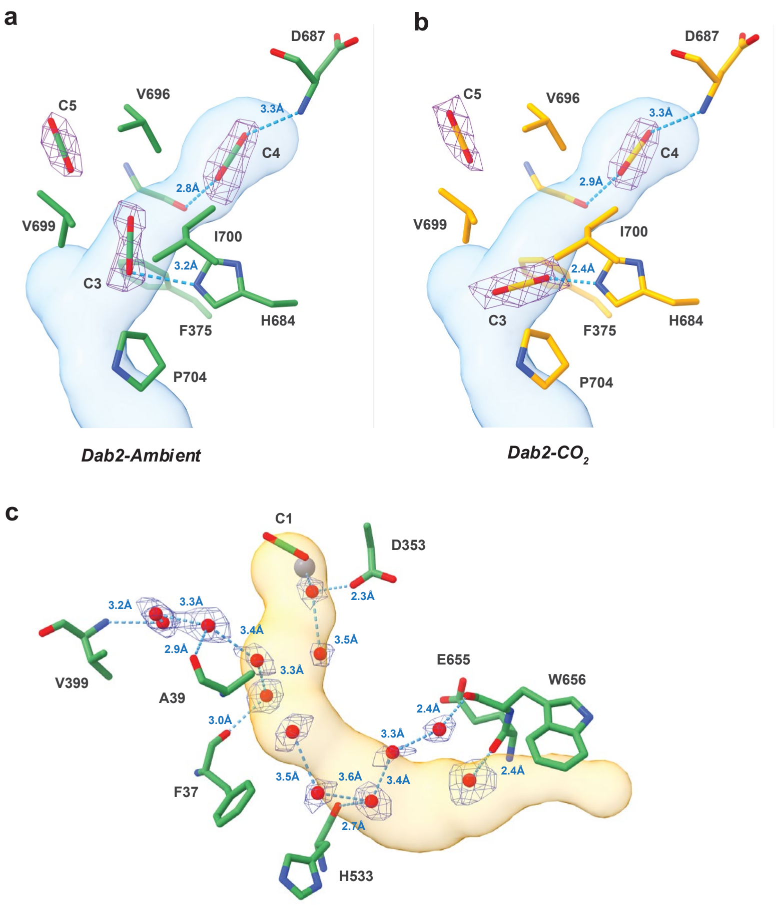

Supplementary Figure 16. Ligands density fitting along the predicted tunnels. Coordination of CO_2 molecules in **a)** *Dab2-Ambient* and **b)** *Dab2-CO₂* within and near the T1 tunnel. Densities are shown at 6 σ. CO_2 molecules are mainly stabilized by sidechain hydrophobic interactions. Blue dashes depict potential hydrogen-bonding with sidechain or backbone amide. **c)** Coordination of *Dab2-Ambient* water molecules in the T2 tunnel. Densities of water molecules are displayed at 7 σ. Blue dash depicted the water molecule bonding network.

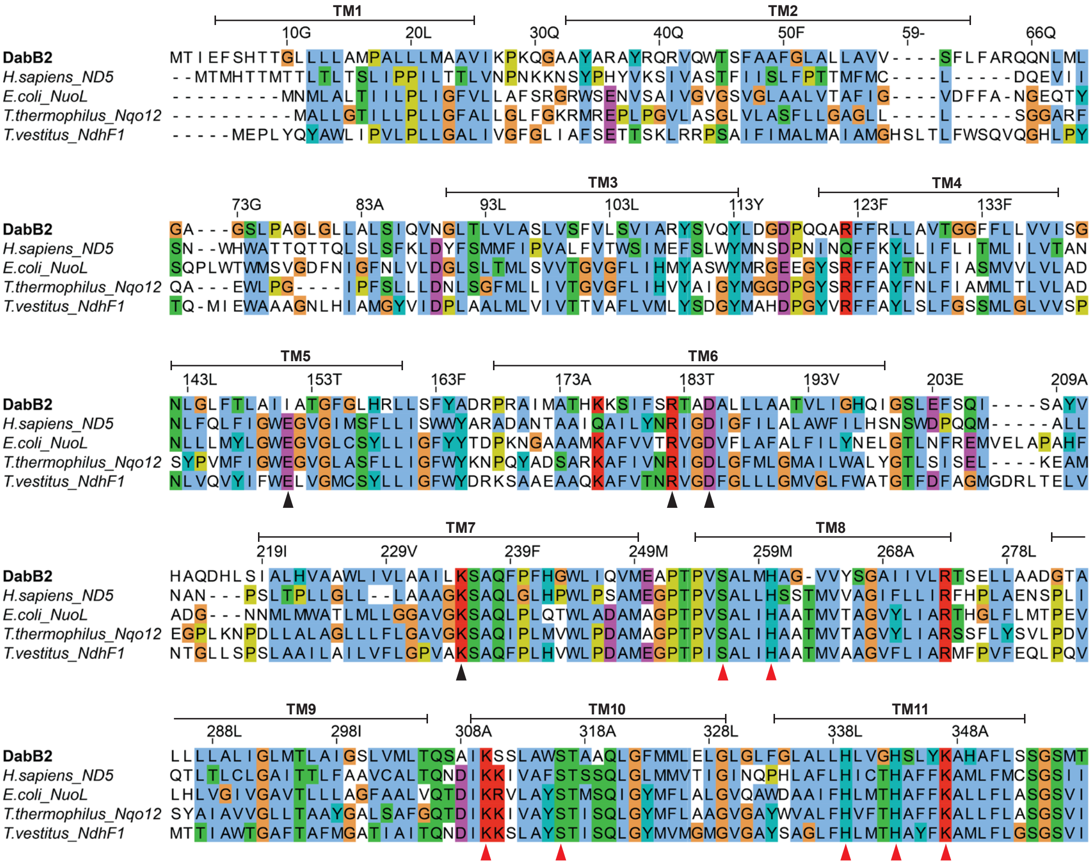

Supplementary Figure 17. DabB2 Multiple sequence alignment. MUSCLE alignment of DabB2 transmembrane helixes TM1 to TM11 on Complex I (-like) distal proton-pumping subunits from *Homo sapiens*, *Escherichia coli*, *Thermus thermophilus* and *Thermosynechococcus vestitus*. Residues are colored by the *Clustal* coloring scheme. Regulatory ion-pairs and residues likely involved in proton transfer are marked by black and red triangle respectively. DabA2 residues number and secondary structure are shown above the alignment.

a

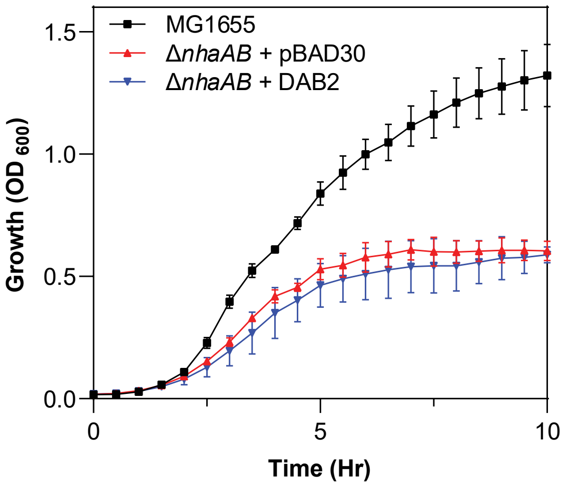

b

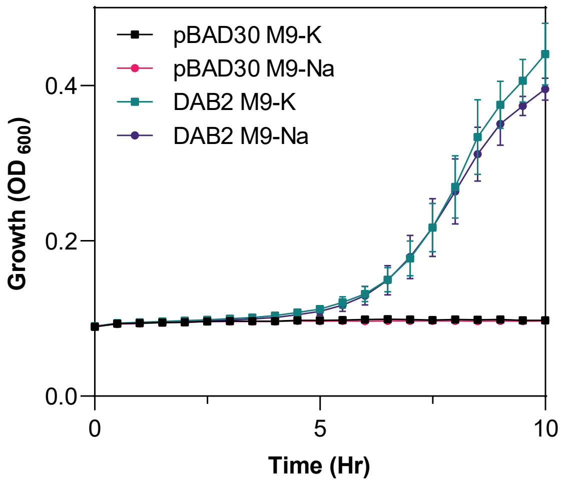

Supplementary Figure 18. DAB2 activity is independent of sodium. a) DAB2 did not restore growth of *E. coli* lacking both NhaA and NhaB sodium transporters under sodium stress (0.1 M). Wild-type *E. coli* MG1655 was used as a positive control. **b)** Complementation of CA deficient *E. coli* by DAB2 in M9 medium prepared with potassium salts (M9-K) or sodium salts (M9-Na). The similar growth profile suggests the complex might not require sodium. Data points and error bars represent means and standard deviations, respectively (n = 4 biological replicates). Source data are provided as a Source Data file.

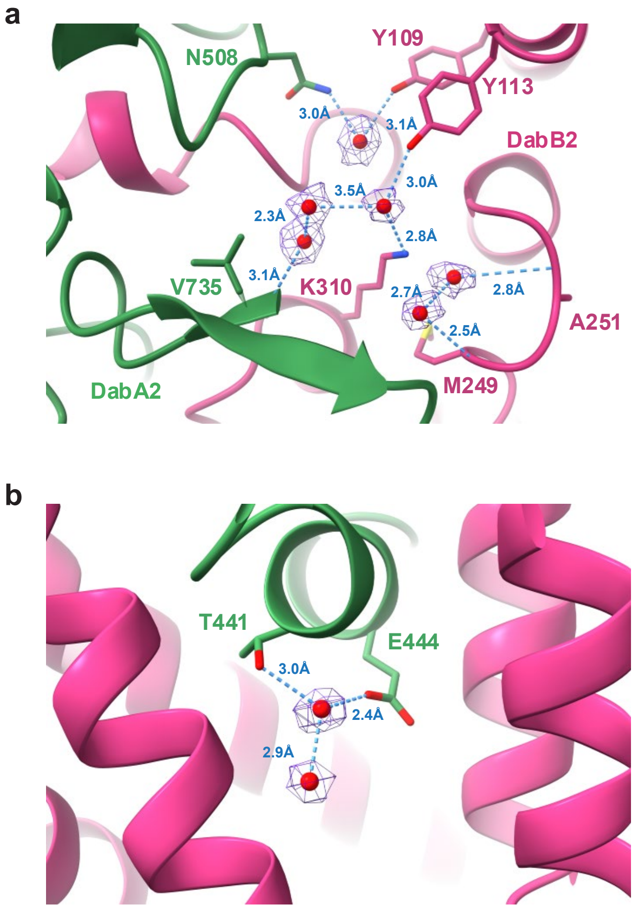

Supplementary Figure 19. Putative proton pathway water molecules. Coordination of the water molecules shown in Fig. 5c at **a)** the periplasmic side and **b)** cytoplasmic side. Densities of water molecules are shown at 7 σ. Blue dashes depict hydrogen-bond with neighboring atoms.

Supplementary Table 1. Cryo-EM data collection, refinement and validation statistics.

	Dab2-Ambient (EMDB-53925) (PDB 9RD0)	Dab2-CO_2 (EMDB-53930) (PDB 9RD9)	Dab2-HCO_3^- (EMDB-53929) (PDB 9RD8)
Data collection and processing			
Magnification	165,000X	165,000X	165,000X
Voltage (kV)	300	300	300
Electron exposure (e–/Å^2)	55	55	55
Defocus range (µm)	-0.5 to -2.25	-0.5 to -2.25	-0.5 to -2.25
Pixel size (Å)	0.73	0.73	0.73
Symmetry imposed	C1	C1	C1
Initial particle images (no.)	6,304,235	2,863,711	3,262,632
Final particle images (no.)	254,703	231,193	226,711
Map resolution (Å)	2.64	2.72	3.22
FSC threshold	0.143	0.143	0.143
Map resolution range (Å)	2.3-3.0	2.4-3.1	2.8-4.0
Refinement			
Model resolution (Å)	2.6	2.7	3.2
FSC threshold	0.143	0.143	0.143
Map sharpening *B* factor (Å^2)	-120.1	-115.1	-90.9
Model composition			
Non-hydrogen atoms	10,687	10,539	10,342
Protein residues	1,320	1,317	1,312
Ligands	10	10	7
B factors (Å^2)			
Protein (mean)	29.82	45.88	17.24
Ligand (mean)	36.46	53.51	35.48
R.m.s. deviations			
Bond lengths (Å)	0.002	0.003	0.005
Bond angles (°)	0.456	0.514	0.963
Validation			
MolProbity score	1.35	1.38	1.59
Clashscore	5.00	6.11	6.62
Poor rotamers (%)	0.28	0.38	0.29
Ramachandran plot			
Favored (%)	97.56	97.78	96.54
Allowed (%)	2.37	2.14	3.30
Disallowed (%)	0.08	0.08	0.15

Supplementary Table 2. Polar interactions between DabA2 and DabB2 identified by the PISA server.

Hydrogen bond

DabB2	Distance (Å)	DabA2	DabB2	Distance (Å)	DabA2
Tyr109	3.44	Trp649	Ser356	3.17	Asn530
Tyr109	3.35	Asn530	Thr358	3.39	Gly738
Gln112	3.17	Gln651	Asp359	2.72	Arg534
Asp115	2.71	Arg653	His360	2.66	Ser464
Arg167	3.06	Glu744	Arg362	2.80	Gln545
Gln247	3.27	Asn746	Arg362	2.81	Arg387
Met249	2.98	Asn746	Gln363	3.28	Arg387
Glu250	3.01	Asn746	Ala364	2.55	Gln423
Thr253	2.93	VAL731	Lys365	2.96	Glu390
Leu304	3.17	Leu154	Lys368	3.44	Gly389
Leu304	2.85	TRP155	Glu431	2.82	His420
Ser307	2.92	Ala145	Gln490	2.62	Ser435
Ser307	2.97	Gly737	Gln490	3.23	Ala436
Ser307	3.17	Asp149	Gln490	2.78	Ala437
Ile309	3.46	Asn734	Asn510	3.37	Asn146
Lys310	3.04	Asn734	Asn510	2.64	Gln142
Gln320	2.95	CYS439	Asn511	3.48	Ser178
His349	2.78	Asp457	Asn511	3.05	Gln142
Leu352	3.26	Asn530	Asn511	3.53	Asn176
Gly355	3.41	Asn530	Arg521	3.16	Ala181

Salt bridge

DabB2	Distance (Å)	DabA2	DabB2	Distance (Å)	DabA2
Arg169	3.79	Glu134	Asp359	3.24	Arg534
Arg169	3.71	Glu138	Asp359	2.72	Arg534
His349	2.78	Asp457	Asp359	2.82	Arg534
Arg167	3.57	Glu744	Glu431	3.47	His420
Arg167	3.88	Glu744	Glu431	2.82	His420
Arg167	3.62	Glu744	Glu500	3.91	Arg171
