# Supplementary material for: Structural basis of membrane potential coupled vectorial CO₂ hydration by the DAB2 complex in chemolithoautotrophs
Source: Nat Commun. 2026 May 5;17:4071. doi: 10.1038/s41467-026-72558-7 (PMC13144422; doi:10.1038/s41467-026-72558-7)
Supplement: Supplementary file 2 — Description of Additional Supplementary Files [file 41467_2026_72558_MOESM2_ESM.pdf]

Description of Additional Supplementary Files

Supplementary Data 1. List of oligonucleotides

Supplementary Data 2. AlphaFold predicted model of the wild-type DAB2 complex
